# Implementation of ML Flow for leprosy contacts in Brazil: Opportunities, pitfalls, and safeguards

**DOI:** 10.1371/journal.pntd.0014238

**Published:** 2026-04-21

**Authors:** Carolina Talhari, Claúdia dos Passos Farias, Helio Amante Miot, Sinesio Talhari

**Affiliations:** 1 Programa de Pós-Graduação em Ciências Aplicadas à Dermatologia, Universidade do Estado do Amazonas, Manaus, AM, Brazil; 2 Fundação Hospitalar Alfredo da Matta de Dermatologia, Manaus, AM, Brazil; 3 Departamento de Dermatologia da FMB-UNESP, Botucatu, São Paulo, Brazil; University of Bremen: Universitat Bremen, GERMANY

## Abstract

Brazil has the second-highest leprosy burden worldwide, with approximately 20,000 new cases reported annually, many diagnosed with advanced disease and disability. To support earlier detection, the Ministry of Health recently approved the ML Flow rapid test for contact evaluation. ML Flow detects immunoglobulin M antibodies against phenolic glycolipid-I of *Mycobacterium leprae*, can be performed at the point of care using finger-prick blood, and yields results within minutes. ML Flow offers important operational advantages. It enables same-visit counseling and may support risk-stratified household-contact follow-up. However, its value is context-dependent. Because anti-phenolic glycolipid-I immunoglobulin M responses correlate with bacillary burden, positivity is more frequent in multibacillary disease, whereas many paucibacillary and pure neural cases have absent or low antibody levels. A seronegative result therefore does not exclude disease. Among asymptomatic contacts, seropositivity varies widely across studies, and in previously treated individuals antibodies may remain detectable for years. Available evidence suggests that seropositive contacts are at increased risk of incident leprosy, although predictive performance varies across settings. Serology alone is not diagnostic, and misuse may lead to stigma, anxiety, unnecessary referrals, and diversion of resources. The contribution of ML Flow is therefore implementation-dependent. In settings with standardized counseling, scheduled re-examination, and reliable referral pathways, it may support risk-stratified follow-up. In settings where these elements are weak, benefits may be limited. Brazil offers an important programmatic setting in which to evaluate this strategy, but only with safeguards: integration with dermato-neurological examination, clear protocols stating that seropositivity is not diagnostic, structured follow-up pathways, quality-controlled training, and systematic recording in the Brazilian Unified Health System information systems. Under these conditions, ML Flow may contribute to earlier diagnosis and disability reduction; without them, it risks adding workload without improving care.

Brazil reports the second-highest number of new leprosy cases worldwide, with approximately 20,000 diagnoses annually. Most cases were multibacillary (MB; 82.4%) and 11.2% presented with grade 2 disability. Delays in case detection/diagnosis can sustain transmission and are associated with an increased risk of nerve impairment and disability at diagnosis, highlighting the importance of timely screening of high-risk groups, such as household contacts [1–4].

This Viewpoint is based on a narrative review anchored in current Brazilian Ministry of Health (BMH) guidance for leprosy contacts and informed by selected PubMed-indexed studies and illustrative international experiences.

In this context, BMH issued Technical Note no. 3/2023, defining a primary care workflow for household-contact screening and follow-up and recommending ML Flow as a qualitative adjunct after clinical examination to support risk stratification and triage. ML Flow is performed only after dermato-neurological screening of household contacts without clinical signs of leprosy. A seronegative result triggers counseling on signs/symptoms and self-examination, with instructions to seek care if symptoms develop. A seropositive result should be communicated as a serologic finding consistent with subclinical infection and higher risk, not as a diagnosis. Bacillus Calmette-Guérin (BCG) eligibility should then be assessed according to vaccination history, and asymptomatic contacts should enter annual surveillance for five years, undergoing clinical reassessment and repeat ML Flow at each annual visit (Fig 1) [4,5].

**Fig 1:**
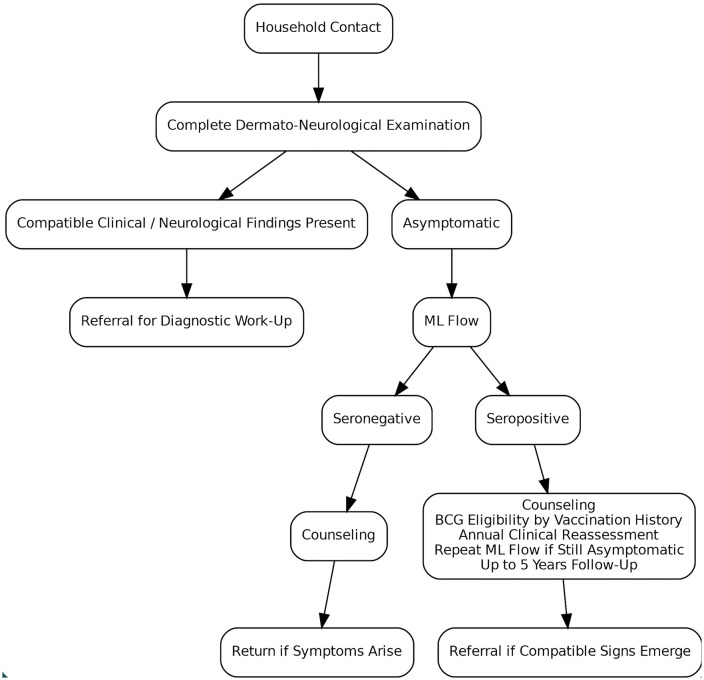
Recommended primary-care workflow for ML Flow use in household contacts under the Brazilian Ministry of Health framework.

Because household contacts are at a high risk of leprosy [6], ML Flow detection of anti-*Mycobacterium leprae* PGL-I IgM is therefore intended to support risk stratification after dermato-neurological examination and to guide follow-up and/or confirmatory evaluation, not to serve as a standalone diagnostic test [4,5]. Any contribution to earlier case detection is indirect: prioritizing seropositive contacts for scheduled reassessment increases opportunities to detect early clinical signs and trigger diagnostic work-up when warranted, rather than by diagnosing leprosy in the absence of signs. Anti-PGL-I IgM seropositivity in asymptomatic contacts should be interpreted as a marker consistent with higher risk, not a diagnosis. Accordingly, it should guide structured follow-up rather than treatment or confirmatory testing based on serology alone [2–6].

ML Flow is a lateral-flow immunochromatographic assay that detects IgM antibodies against the *M. leprae* PGL-I antigen. Under the BMH Technical Note, it is implemented as a qualitative serologic test, with results interpreted in this manuscript as anti-PGL-I IgM seropositive or seronegative. Because results are read visually within a defined time window, interpretation, particularly of faint lines, can vary between operators and requires standardized training and basic quality assurance. In the Brazilian program, this corresponds to the Fast ML Flow Hanseníase rapid test (Bioclin, Brazil; derived from the original ML Flow), distributed within the public health network for clinical use [4,5]. In some settings, anti-PGL-I serology is performed using quantitative or instrument-read platforms, including enzyme-linked immunosorbent assay (ELISA) and reader-based formats, which differ operationally from visually interpreted point-of-care assays [7]. Although platforms differ, the broader interpretive principles and implementation safeguards discussed here remain relevant to anti-PGL-I IgM testing among contacts in different epidemiological contexts. This Viewpoint focuses on the qualitative point-of-care rapid test used at scale for routine household-contact surveillance in Brazil [4,5,7–9].

In patients with clinical leprosy, reported diagnostic performance varies according to clinical form and epidemiological setting. Anti-PGL-I IgM assays have shown high sensitivity in MB disease across endemic settings (reported range ≈75–97%), whereas sensitivity is substantially lower in paucibacillary (PB) disease in Brazilian data (≈32%) [6,7,10–12]. Among asymptomatic contacts, reported anti-PGL-I seropositivity also varies across studies (~2% to >20%) and is influenced by local endemicity, contact type, the MB/PB profile of the index case, methodological factors, and follow-up intensity (Table 1) [13–15].

**Table 1:** Operational interpretation of anti-PGL-I IgM serology across key populations relevant to ML Flow implementation in Brazil.

Population	Expected anti-PGL-I IgM serology	What a seropositive result may mean	What it does not mean	Programmatic action
**Multibacillary (MB) clinical leprosy**	Often seropositive; sensitivity generally higher than in PB disease	Higher bacillary burden; serology may be concordant with clinically evident disease	Does not establish diagnosis on its own or replace clinical classification	Use only as an adjunct; diagnosis/classification remains clinical, bacteriological, and programmatic
**Paucibacillary (PB) clinical leprosy**	Frequently seronegative; sensitivity is substantially lower	If seropositive, may reflect antibody response in a subset of cases	A seronegative result does **not** exclude PB disease	Do not use seronegativity to reassure or defer work-up when clinical suspicion is present
**Pure neural leprosy/ early disease**	Often seronegative or low-level response expected	Occasional seropositivity may occur, but is not diagnostic	Seronegativity does **not** exclude neural involvement or early disease	Any compatible neurological findings should prompt referral/diagnostic work-up regardless of serology
**Asymptomatic household contact, seropositive**	Variable across studies/settings	Serologic marker consistent with subclinical infection and associated with higher risk	Does not diagnose leprosy and should not trigger treatment by itself	In Brazil: communicate risk carefully, assess BCG eligibility according to vaccination history, and enter annual clinical surveillance for 5 years, with repeat ML Flow if still asymptomatic.
**Asymptomatic household contact, seronegative**	Common, especially in lower-risk or lower-endemicity settings	Lower measured serologic risk at that time	Does not exclude early disease, PB or pure neural leprosy, subclinical infection, or future disease	In Brazil: counseling on signs/symptoms and self-examination; passive surveillance and return if symptoms develop
**Previously treated leprosy patient**	May remain seropositive for years after treatment	Persistent antibody detection after past disease/treatment	Does not indicate active disease, relapse, or treatment failure by itself	Interpret with caution; clinical assessment remains decisive; avoid using serology alone to infer active disease
**Community members outside targeted contact evaluation**	Context-dependent; background seropositivity may occur	May reflect background anti-PGL-I seropositivity in endemic settings	Does not support diagnosis of active leprosy at population level	ML Flow should not be used for community-wide active case finding or population screening
**Symptomatic contact with compatible skin/neurological findings**	May be seropositive or seronegative	If seropositive, may support risk framing in context	Serology alone neither confirms nor rules out disease	Clinical suspicion should trigger diagnostic work-up/referral (e.g., specialist evaluation, SSS, biopsy and/or PCR as indicated), regardless of serology

Anti-PGL-I IgM serology should be interpreted within a complete dermato-neurological examination and a defined referral pathway. In this Viewpoint, serology is framed as a tool for risk stratification and follow-up planning among household contacts, not as a standalone diagnostic test. Expected serologic patterns and predictive value vary by clinical form, endemicity, contact type, index-case spectrum, assay platform, and follow-up intensity.

Given this performance profile and the heterogeneous distribution of leprosy in Brazil, ML Flow may support targeted evaluation of household contacts. However, its predictive value is context-dependent and is not intended for community-wide screening for active case finding [1,4–6]. In high endemicity areas, high background seropositivity limits the positive predictive value for clinical disease; in very low-prevalence settings, the low base rate (pre-test probability) similarly reduces the positive predictive value, increasing the proportion of seropositive results that do not correspond to clinical disease [4,7,10,15].

When used after a complete dermato-neurological examination, ML Flow may support counseling and risk stratification for follow-up and referrals. However, microbiological tests (e.g., slit-skin smear [SSS], biopsy, and/or polymerase chain reaction [PCR]) are indicated for contacts with cutaneous and/or neurological findings compatible with leprosy and should not be triggered by seropositivity alone, nor deferred because of a seronegative result [4,5].

From a programmatic standpoint, the principal value of ML Flow in asymptomatic contacts is risk stratification and triage for follow-up and/or confirmatory evaluation rather than diagnosis. Key programmatic advantages include [4–7,10–15]:

- Operational efficiency: Enables same-visit counseling and a structured, risk-based surveillance plan, while clinical suspicion (not serology alone) remains the trigger for confirmatory testing and specialist referral.
- Contact-surveillance support: Aggregated seroprevalence data among identified household contacts may help characterize exposure patterns and follow-up needs within high-risk household clusters, but do not inform population-based surveillance.
- Targeted monitoring: Seropositive contacts can be prioritized for follow-up. In the current BMH framework, ML Flow structures counseling, BCG assessment, and longitudinal clinical surveillance of asymptomatic contacts, not eligibility for chemoprophylaxis.

Cohort evidence suggests that seropositive contacts have an increased risk of incident leprosy (summary OR ≈3.1 in a systematic review and meta-analysis) [12]. This supports closer, risk-stratified clinical surveillance. However, sensitivity is limited and predictive performance varies by setting; notably, a longitudinal cohort in Bangladesh found no association between baseline anti-PGL-I antibody levels and subsequent disease onset [13]. Comparative evidence that risk-stratified active follow-up of seropositive asymptomatic contacts accelerates diagnosis beyond high-quality counseling for all contacts remains limited [12–14]. Under the current Brazilian framework, asymptomatic seropositive contacts enter annual follow-up for five years. This creates an opportunity to evaluate predictive value and programmatic impact prospectively, although a formal evaluation framework has not yet been specified [4,5]. Accordingly, ML Flow should be framed primarily as an implementation tool to structure reassessment and referral pathways rather than as a standalone strategy to ‘speed up’ diagnosis.

ML Flow has important limitations that should inform policy and practice, each with corresponding safeguards and program actions discussed below [4–7,10–15]:

- Low applicability in PB and pure neural forms: A seronegative result cannot exclude early disease, PB or pure neural leprosy, as these presentations often lack detectable anti-PGL-I IgM. A seronegative result must never be used to downgrade clinical suspicion, defer diagnostic work-up, or delay referral when compatible cutaneous and/or neurological signs or symptoms are present; such contacts should be evaluated and, when indicated, undergo confirmatory testing and/or referral regardless of serology.
- Persistent seropositivity: Antibodies can remain detectable for years after treatment, risking misinterpretation in previously treated individuals.
- Risk of overdiagnosis and stigma: Labeling asymptomatic seropositive contacts as “patients” can cause unnecessary treatment and psychosocial harm. Seropositive results without diagnostic criteria may also cause stress during prolonged surveillance. Under the BMH Technical Note, asymptomatic seropositive contacts undergo annual surveillance for up to five years with clinical reassessment and repeat ML Flow if still asymptomatic.
- Operational vulnerabilities: Inadequate training, inconsistent line interpretation and supply-chain interruptions can compromise accuracy and trust.
- Uneven availability: As the test is absent from most primary care units, inconsistent protocols limit epidemiological monitoring.
- Bias toward clinically evident MB cases: Performance is best in MB disease, where signs are already obvious. This raises doubts about the incremental value for earlier detection, particularly for PB and pure neural forms.

Illustrative experiences from other countries reinforce this concern, but do not imply policy equivalence across national programs. In Bangladesh and Nepal, over-reliance on serology without robust follow-up systems may lead to unnecessary referrals, while in India, the added benefit was minimal where surveillance was weak [6,11–13]. Where programs deliver standardized counseling, scheduled re-examination, and reliable referral pathways, serology helped identify higher-risk contacts for active surveillance, thereby increasing opportunities to detect early clinical signs and trigger diagnostic work-up when indicated. Where these elements were weak, added workload brought little measurable public health benefit and could increase anxiety and stigma if results are misinterpreted and follow-up/referral pathways were unreliable [11–14].

To ensure ML Flow strengthens rather than undermines control efforts, safeguards should directly address the limitations outlined above, linking each to a practical program response [4–7,10–15]:

- Integration with clinical evaluation: Perform testing only after a full dermato-neurological examination, with protocols stating that seropositivity does not equate to diagnosis. At minimum, the primary-care dermato-neurological examination should include inspection for compatible skin lesions; simple sensory testing of suspicious lesions and symptomatic areas; palpation of accessible peripheral nerves for thickening or tenderness; and brief screening for sensory symptoms, weakness, or neuropathic complaints. Any compatible cutaneous lesion with sensory change, peripheral nerve thickening/tenderness, focal sensory loss, motor deficit, or indeterminate findings should prompt referral for diagnostic work-up and/or specialist evaluation, regardless of serology.
- Referral pathways: Per Technical Note no. 3/2023, asymptomatic seropositive contacts enter annual surveillance for up to five years, with repeat rapid testing if they remain asymptomatic; contacts with symptoms/signs or indeterminate assessment should be referred for diagnostic work-up (e.g., SSS and specialist evaluation) as indicated.
- Stopping rules and exit from active follow-up: Under the current Brazilian framework, asymptomatic seropositive contacts undergo annual clinical reassessment for five years, with repeat rapid testing if they remain asymptomatic. Because the national guidance does not explicitly define post-surveillance exit rules, programs should avoid indefinite repetition of testing based on serology alone. If no compatible cutaneous or neurological signs emerge during the defined surveillance period, continued follow-up should be guided by clinical and programmatic judgment rather than serology alone; counseling at the end of follow-up should reinforce return instructions if symptoms arise and avoid language that may increase anxiety or stigma.
- Standardized counseling: Communicate that seropositivity is consistent with subclinical infection and higher risk, but diagnosis requires compatible clinical signs and, when indicated, confirmatory testing or structured follow-up.
- Training and quality control: Reference centers should provide competency-based training and regular quality assessments, including correct test execution, adherence to the read-time window, interpretation of faint bands, recognition of invalid tests, standardized documentation, and periodic refresher training.
- Data integration: Record test requests and results in the appropriate national information systems and ensure confirmed leprosy cases are notified in the Sistema de Informação de Agravos de Notificação (SINAN), to support monitoring of coverage and subsequent case detection.
- Implementation success metrics: Programs should assess whether ML Flow is adding value by monitoring key indicators such as the proportion of household contacts receiving complete dermato-neurological examination, referral completion, new-case detection among followed contacts, grade 2 disability at diagnosis among contact-detected cases, counseling fidelity, and loss-to-follow-up. These indicators help determine whether ML Flow is strengthening contact evaluation and earlier clinical detection rather than simply increasing testing volume.

Brazil’s established leprosy contact-surveillance pathways within the Brazilian Unified Health System support ML Flow; however, programmatic value depends on consistent safeguards, standardized counseling, clear referral criteria and quality assurance to avoid unnecessary procedures and prolonged follow-up [4,5]. When implemented in accordance with BMH Technical Note no. 3/2023 and supported by trained personnel and robust follow-up/referral pathways, ML Flow could help achieve earlier diagnosis, reduce disability, and contribute to interrupting transmission [4,5]. Used indiscriminately, however, it risks misdirecting resources, generating unnecessary referrals, and eroding community trust.

Although this Viewpoint focuses on the qualitative anti-PGL-I IgM rapid test specified in current BMH guidance, next-generation quantitative and multi-biomarker point-of-care assays are under development and may improve performance [8,9]. If adopted programmatically, their incremental value would still depend on the same implementation safeguards emphasized here.

A coordinated global effort is needed to define how anti-PGL-I rapid tests should be integrated into contact-management pathways, with clear action thresholds and safeguards against misinterpretation. In Brazil, ML Flow should be used only as a qualitative adjunct for targeted household-contact evaluation after a complete dermato-neurological examination, to support counseling and risk-stratified, time-bound follow-up within contact-surveillance pathways. In individuals without dermato-neurological signs, seropositive results should prompt structured clinical follow-up rather than diagnosis or treatment decisions based on serology alone. ML Flow should not be used as a standalone diagnostic test, a rule-out test, a trigger for confirmatory testing in asymptomatic contacts solely on serology, or a tool for community-wide active case finding; likewise, a seronegative result must not downgrade clinical suspicion or delay referral. In practice, programmatic impact will depend as much on implementation quality and user capacity as on test characteristics.
